# Multi Year Observations Reveal Variability in Residence of a Tropical Demersal Fish, *Lethrinus nebulosus*: Implications for Spatial Management

**DOI:** 10.1371/journal.pone.0105507

**Published:** 2014-09-02

**Authors:** Richard D. Pillans, Douglas Bearham, Andrew Boomer, Ryan Downie, Toby A. Patterson, Damian P. Thomson, Russel C. Babcock

**Affiliations:** 1 CSIRO Marine and Atmospheric Research, Brisbane, Queensland, Australia; 2 CSIRO Marine and Atmospheric Research, Hobart, Tasmania, Australia; 3 CSIRO Marine and Atmospheric Research, Floreat, Perth, Western Australia, Australia; 4 SIMS/IMOS Animal Tagging and Monitoring, Mosman, New South Wales, Australia; Leibniz Center for Tropical Marine Ecology, Germany

## Abstract

Off the Ningaloo coast of North West Western Australia, Spangled Emperor *Lethrinus nebulosus* are among the most highly targeted recreational fish species. The Ningaloo Reef Marine Park comprises an area of 4,566 km^2^ of which 34% is protected from fishing by 18 no-take sanctuary zones ranging in size from 0.08–44.8 km^2^. To better understand Spangled Emperor movements and the adequacy of sanctuary zones within the Ningaloo Reef Marine Park for this species, 84 Spangled Emperor of a broad spectrum of maturity and sex were tagged using internal acoustic tags in a range of lagoon and reef slope habitats both inside and adjacent to the Mangrove Bay Sanctuary zone. Kernel Utilisation Distribution (KUD) was calculated for 39 resident individuals that were detected for more than 30 days. There was no relationship with fish size and movement or site fidelity. Average home range (95% KUD) for residents was 8.5±0.5 km^2^ compared to average sanctuary zone size of 30 km^2^. Calculated home range was stable over time resulting in resident animals tagged inside the sanctuary zone spending ∼80% of time within the sanctuary boundaries. The number of fish remaining within the array of receivers declined steadily over time and after one year more than 60% of tagged fish had moved outside the sanctuary zone and also beyond the 28 km^2^ array of receivers. Long term monitoring identified the importance of shifting home range and was essential for understanding overall residency within protected areas and also for identifying spawning related movements. This study indicates that despite exhibiting stable and small home ranges over periods of one to two years, more than half the population of spangled emperor move at scales greater than average sanctuary size within the Ningaloo Reef Marine Park.

## Introduction

The cumulative behaviour of individuals in determining net population movement patterns has significant implications for sustainable management of harvested species. Individual animal movements are particularly important for both spatial management and Ecosystem Based Fisheries Management (EBFM) which are increasingly used as means of preserving biodiversity, maintaining habitat structure and ecosystem function, and for preservation of genetic diversity [1], [2], [3]. While ecologically fundamental, observing, understanding and predicting an animal's habitat requirements has been difficult. The difficulties in observing the movements of marine animals have been addressed by technological developments in the field of animal telemetry [4]. For reef fish the use of implanted acoustic tags has been particularly significant [5], [6]. This study demonstrates the importance of long term monitoring and the need to consider movement at multiple spatial and temporal scales in determining the spatial usage in a reef fish population.

Marine spatial management, whether for conservation or EBFM, is complex because the effectiveness of any spatial management measure will be a function of the size of spatial management units and the scale of movement of the species in question. The proportion of populations that will be protected, by no-take MPAs for example, is likely to be positively correlated with reserve size and vary inversely with the species mobility [7], [8], [9], [10]. Consequently while there is a clear expectation that some effect of protection on exploited species will be offered by no-take zones, it is highly uncertain which species will respond, or to what extent, resulting in uncertainty around the effectiveness of no-take areas in protecting multiple species. Variability both in the effectiveness of MPA protection and any benefits to fisheries via spillover are likely to be a function of variability in individual movement patterns and habitat requirements combined with the size, shape and habitat encompassed by the MPA [9], [11], [12], [13], [14], [15], [16].

Marine Protected Areas (MPAs) and spatial management are both important tools for Ecosystem Based Fishery Management (EBFM) and fisheries management [1], [2], [17], [18]. There is mounting evidence for long term positive conservation outcomes within no-take MPAs with increases recorded in the biomass, density, individual size and species richness in the majority of studies irrespective of study latitude and reserve size (see [2], [19], [20]). In addition to the benefits for single species which gain protection from MPAs there is also growing evidence for MPAs restoring ecosystem function through cascading trophic interactions resulting from protection of target species (see [3]). Although the evidence for spillover is less well documented, due to the complexity associated with its measurement, advances in telemetry and stable isotope technology have allowed some studies to document spillover of adults and larvae into surrounding non-reserve areas [20], [21], [22](Goñi *et al.* 2006, Lester *et al.* 2009, Harrison et al. 2012).

In order to deliver any of their potential benefits MPAs need to afford long-term protection of species in order to maintain populations of large, highly fecund individuals. If MPAs are too small, resident fish that roam into adjacent areas or that have multiple home ranges will be more susceptible to capture [5], [8], [13], [23]. The majority of reef fish species studied to date have shown limited movement [5], [24], [25], [26], [27]but also large variability in home range. Parrotfish, surgeonfish and goatfish tagged in Hawaii demonstrated movements from 0.1–0.6 km with a very few individuals moving up to 2.0 km from the tagging site across sand channels between reef habitats [5].In New Caledonia, parrotfish and coral groupers moved up to 5.0 km from the tagging site and crossed areas of sand channel between reefs [25]. Within Australia, few studies have been conducted in coral reef environments. Coral trout *Plectropomus leopardus* demonstrated limited movement up to 250 m outside the spawning season [24] and larger movements up to 5.2 km from established home range during spawning periods [28]. More recent work on short term movement of steephead parrotfish *Chlorurus microrhinus* on the Great Barrier Reef has demonstrated movements of less than 400 m from the point of capture [27].

While individual variations in habitat use or movement and mobility may be of intrinsic ecological interest, management of fish species is pursued at the level of populations. It is therefore the cumulative behaviour of individual fish and how they scale to overall population level movement patterns that has significant implications for sustainable management and/or conservation of fished species. Despite the importance of movement, very few studies have addressed population level movement of a number of animals from different age classes tagged in a variety of habitats [16], and none in a coral reef environment.

The Spangled Emperor *Lethrinus nebulosus* (Försskal, 1775) is widespread throughout the Indo-West Pacific, including the Red Sea, Persian Gulf, East Africa to Southern Japan and Samoa [29]. It occurs in nearshore and offshore areas and in coral and rocky reefs, coralline lagoons, seagrass beds, mangrove swamps, and coastal sand and rock areas to depths of at least 75 m [29]. Adults are found alone or in small schools, whereas juveniles form large schools. It feeds mostly on echinoderms, molluscs, crustaceans, and to a lesser extent on polychaetes and fishes [29]. This species has a non functional protogynous hermaphroditic life history strategy [30] that prevents size-based sex determination.

Within the Ningaloo Reef Marine Park (NRMP), *L. nebulosus* is an important predator of grazing invertebrates along the Ningaloo Reef, and is likely to play an important role in maintaining ecosystem function [31]. A creel survey of recreational fishers in the NMP showed that members of the family Lethrinidae were the most targeted species [32] and this, combined with their longevity and slow growth along the west Australian coastline [30], [33], makes them susceptible to over-exploitation [34], [35]. Indeed, despite only being captured by recreational fishers within the NMP, there is evidence of population decline as well as a reduction in the modal age and percentage of old fish [33] along the Ningaloo coastline. During the period in which this decline occurred, the area of no take MPA's in the Ningaloo Marine Park was increased from approximately 10% to 34% [36].

Conventional mark-recapture tagging of lethrinids within the Ningaloo Marine Park supports the theory of limited movement. Moran et al. [37] tagged 1781 *L. nebulosus* and *L. atkinsoni* with 60 fish recaptured over three years. Of these recaptures, 66% were made within 5.5 km of the tagging site, and 75% within 10 km, even in the third year after tagging. A few fish had moved 110 km within three months of the release and no fish were recaptured more than 148 km away. Almost all recaptures occurred within the lagoon (Ross Marriott, Pers. Comm.) with the recapture data supporting the theory that most individuals within the lagoon have a relatively small area of occupancy, even over long periods at liberty. However, the spatial resolution of these data is coarse (11.1 km), especially in relation to the average size of no-take zones at NMP (mean linear extent 9.0 km).

Using an array of acoustic receivers and surgically implanting acoustic tags into 84 *L. nebulosus*, the primary objective of this study were to 1) monitor movement patterns of a wide size range of *L. nebulosus* captured in different habitats within the Ningaloo reef and determine the site fidelity and size of activity centres (“home range”) of individuals, 2) using estimates of home range investigate variability in habitat usage and residency relevant to population level movement patterns and 3) using movement characteristics, assess the adequacy of MPA zoning within the NRMP.

## Methods

### Study site

**Figure 1:**
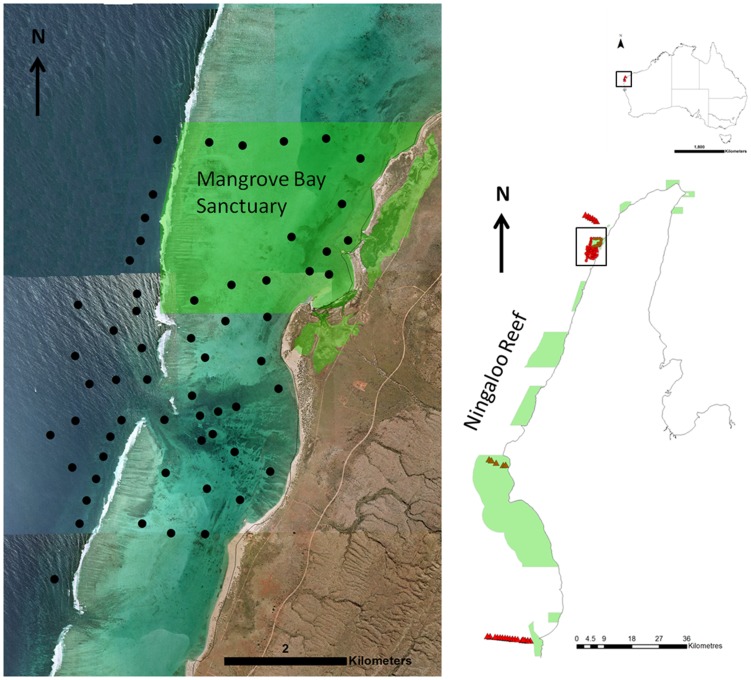
Map of study area. Map of study area with the red triangles showing the lines of cross shelf receivers. From north to south: Tantabiddi, Coral Bay and Winderbandi Point. The Coral Bay receivers covered both lagoon and reef slope habitat. Sanctuary zones in the Ningaloo Marine Park are shown as light green shading. The black circles show the acoustic receivers within and adjacent to the Mangrove Bay Sanctuary.

The Ningaloo Reef Marine Park (NRMP) encompasses Australia's largest fringing reef which is one of the world's largest fringing reefs [36], covering a total area of 4,566 km^2^. It runs along 300 km of Western Australia's coastline from Bundegi in the Exmouth Gulf to Red Bluff in the south (Figure 1). In 2006 the NMP was substantially extended and 34% of total Park area was incorporated into limited or no-take Sanctuary Zones under the revised Management Plan (2005–2015). Areas where no fishing is allowed are referred to as sanctuary zones. The NRMP is zoned for multiple uses and although no commercial fishing is allowed fishing is permitted in recreation zones with recreational fishing catch controlled by possession and size limits. Although areas of protection were chosen based on the best available knowledge and with a view to including the full spectrum of representative marine habitats, many of these decisions were made without in-depth knowledge of the biological communities that reside there and without data on the movement patterns of any fish species.

### Acoustic monitoring system

An array of acoustic receivers was located within and adjacent to the Mangrove Bay sanctuary zone (695 ha) and extended from <1 m of water near the shoreline to the reef slope in ∼50 m of water (Figure 1). Receivers were spaced 200–800 m apart and detection ranges generally did not overlap. The array encompassed habitats including mangrove-lined shores, limestone pavement, coral reefs interspersed with expanses of sand as well as areas of flat hard substratum dominated by macroalgae (predominantly *Sargassum* and other fucalean algae) within the lagoon. A continuous fringing reef creates a barrier to movement out of the lagoon at low tide and during times of high swell however a reef pass provides direct access to deeper reef slope waters. Several large *Porites*-dominated patch reefs are present in the reef pass. The reef slope consists of spur and groove habitat as well as areas of limestone reef interspersed with sand patches. Beyond 35 m, the substratum is predominantly sandy sediment with occasional flat limestone reef.

An array of Vemco VR2 and VR2W acoustic receivers was used to monitor the movements of tagged fish. The Mangrove Bay array consisted of 50 acoustic receivers from December 2007–May 2008 and 60 acoustic receivers from May 2008–May 2010. In addition to the Mangrove Bay array, there were three cross shelf lines of receivers extending from the reef slope (∼12 m) to the 200 m isobath located along the Ningaloo Reef (Figure 1).

Individual *L. nebulosus* were internally tagged with Vemco coded transmitters (tags). Depending on fish size, individuals were tagged with a V9-2L, V9-2H, V13-1H or V16-4H transmitter. These transmitters range in size from 9×29 mm to 16×68 mm and weighed between 2.9–26 g in water. The pulse rate of transmitters varied from 40–320 s and battery life varied from 185–2020 d depending on the frequency of each ping and the power output of the tag. Each successfully decoded pulse train was recorded as a single detection in the memory of the individual VR2 as the transmitter's identification number, date and time. Receivers were downloaded every three to four months throughout the study, and the batteries were changed at least every six months.

**Figure 2:**
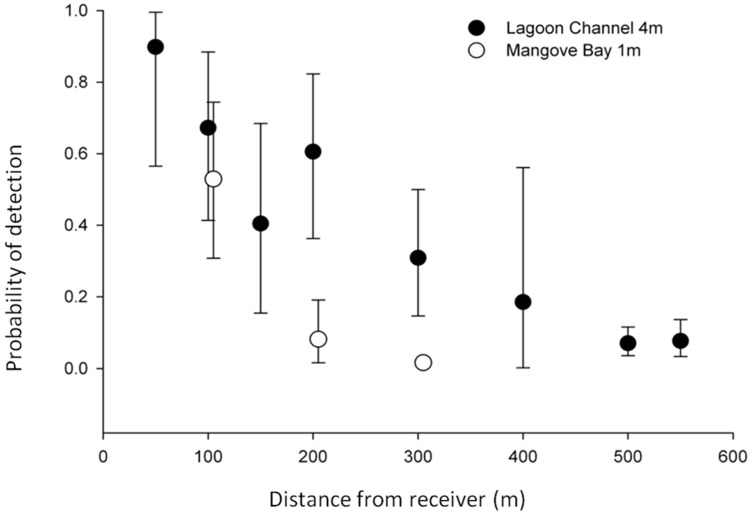
Mangrove Bay array range testing. Proportion of detections received by VR2 receivers at increasing distances from the test transmitter in the lagoon channel (filled circles, 4.0 m water depth) and at Mangrove Bay (white circles, 1.0 m water depth).

Range tests were done by placing one of each of the four transmitter types used at 50, 100, 150, 200, 300, 400 and 500 m away from individual receivers in a straight line. This was also done in at least two directions more than 90° apart on 20 receivers within the array. Range testing was conducted in a variety of wind conditions ranging 0–30 knots. These data were used to determine a probability of detection within the array (Figure 2).

### Capture and tagging

Capture and tagging of fish was conducted under CSIRO Brisbane Animal Ethics Permit (permit A2/07). Fish were captured with rod and line between November 2007–November 2009. Fish chosen for tagging were placed in a 120 l tub containing 30 mg.l^−1^ of AQUI-S in seawater. Fish remained in the tub until they reached stage III anaesthesia at which time they were placed on their dorsal surface into a V-shaped piece of foam lined with plastic. After removing a few scales, a small incision was made slightly off the mid-line between the pelvic fins and anus. Transmitters that had been soaking in an antiseptic bath (povidone iodine and distilled water 5∶100) for at least 30 min were then inserted into the peritoneal cavity. Depending on the transmitter size, either two or three dissolving sutures were used to close the wound. Following surgery, fish were measured and externally tagged with a plastic dart tag (Hallprint, South Australia). A mass dependent dose of ENGEMYCIN, (100 mg Oxytetracycline.ml^−1^) was administered intramuscularly in the dorsal surface. Fish were allowed to recover in a 120 l tub filled with continuously replenished seawater. The average time from capture to completion of surgery was six to seven minutes while recovery times varied ranged from 10–30 min. Once fully recovered, fish were released at the site of capture.

### Detection and spatial analysis

The detection span of each individual was calculated as the date from first detection to last detection whereas days detected was the total number of days on which each individual was detected. Kernel distribution was calculated for those animals that were detected for more than 30 days and on at least one receiver. Area utilisation was estimated using the utilisation distribution [38] and its estimates with kernel techniques [39]. Utilisation distribution is a probability density function that quantifies an individual's relative use of space [40]. It depicts the probability of an animal occurring at a location within its home range as a function of relocation points (data obtained from receiver detections) [41].

Kernel distribution (50 and 95%) was calculated using the Hawth's tools extension for ArcMap. Kernel area was calculated using the animal movement extension for Arcview (ANME). The data from acoustic receivers are not explicitly spatial in the sense that the location of the receiver which detects an individual is obviously not the true location of that individual. However, the frequency of detection at a receiver is directly related to the location of the individual. As a result estimation of a smoothing factor (Hlscv) from treating acoustic detection data identically to continuous tracking data (e.g. from a GPS) will greatly underestimate home range size and result in an improbable series of discrete kernels. In our case smoothing factors calculated using ANME in Arcview, were between 100–500 and resulted in discreet kernels around individual receivers. Determining how to address these problems is an open research question for acoustic data. Centre of activity methods (e.g. [42] rely on overlapping receivers and similarly ad hoc treatments of time (choice of time step). Other approaches applied in this area – such as Brownian bridge models (see [43]) also have limitations and bring strong assumptions about the nature of movements between receivers which are difficult to test using the available data.

Given that our primary goal was to examine movement in relation to usage of an MPA, there was a need to apply a suitably precautionary approach. In this instance, and given the likely detection radius established from range testing, this entails the choice of a smoothing factor which will be robust given the detection radius of an animal. A range of smoothing factors from 100–1200 were tested, however, a value of 1000 provided the most realistic estimates of KUD given the detection range and spacing of receivers combined with where animals had been detected. Using this larger smoothing factor allowed for variability in detectability within the array and gaps in the detection radii of individual receivers to be accounted for given that the probability of detection varied considerably (from 0–100%) depending on the distance of a transmitter to a receiver/s within the array. Although higher smoothing parameters will overestimate kernel distributions and also resulted in less variation between animals, it provided a more robust and precautionary assessment of spatial utilisation at a population level as opposed to what may be misleading representations of multiple kernels centred on individual receivers. To evaluate overlap of home range with the Mangrove Bay Sanctuary, the area of 95% kernel inside the sanctuary boundary was calculated for individual fish tagged inside and outside the MPA.

Behaviour at the individual level as characterised by the 50 and 95% kernel density and distance from kernel centre to tagging location were summarised at the population level in order to evaluate movement characteristics, illustrate the scale of movement and habitat use, and the range of variation in behaviour within the population. To obtain an estimate of the distance that fish moved, the maximum distance moved between receivers within the array was measured as the maximum straight line distance between receivers that had detected individual fish. Maximum distance moved was only calculated for fish that were detected on more than one receiver. Although this measure could potentially be biased by the array design, given the length of time fish spent in the array and the number of receivers detecting individuals (Table 1), it provides a measure of scale of detectable movement within the array.

**Table 1:** Tag detection relationships with tag technical specifications and fish biological characteristics.

		*Individual Parameters*						*Overall Regression*		
		Battery Life	Transmission interval (min)	Tag range (avg)	Habitat tagged in	Fork Length (cm)	Distance to array edge	R-square	F Value	p
Total Detections	F *(p)*	3.55 (0.0630)	-	-	-	-	-	0.0415	3.55	0.0630
Span of detections (days)	F *(p)*	11.31 (0.0012)	-	-	-	-	-	0.1212	11.31	0.0012
Days detected (No.)	F *(p)*	-	-	-	-	-	-	-	-	-
Proportion days detected (days/span)	F *(p)*	-	-	-	6.06 (0.0159)	3.21 (0.0767)		0.1390	6.54	0.0023
Tag location to Kernel centre*	F *(p)*	5.47 (0.0242)	5.55 (0.0232)	7.43 (0.0093)	-	-	-	0.1678	2.82	0.0502
Receivers detecting (No.)	F *(p)*	-	-	-	-	13.45 (0.0004)	-	0.1409	13.45	0.0004
Kernel area (50%)*	F *(p)*	-	-	-	28.33 (<.0001)	4.90 (0.0326)	-	0.4223	14.62	<.0001
Kernel area (95%)*	F *(p)*	-	-	-	76.85 (<.0001)	-	-	0.6709	40.78	<.0001

Multiple backward elimination stepwise regression of animal, tag and habitat parameters against detection and movement measurements for 84 *L. nebulosus* tagged and monitored with the Mangrove Bay array. Overall regression R-squared, F and P values represent the overall significance of the combined influence of all factors. Significant interactions between parameters are illustrated by F and P values, - = Non-significant interaction.

* = subset of data above for which kernels could be calculated, distances to kernel centre of >10 entered as 10.

### Temporal analysis

A multiple stepwise backwards elimination regression was used to evaluate the effect of fish size, battery life, transmission interval, tag range, habitat tagged and the shortest distance from tagging location to array edge on detection and movement parameters. Habitats in which fish were tagged included; reef slope, reef flat, coral outcrops within the lagoon, Mangrove Bay and shoreline pavement. Movement parameters included total number of detections, number of receivers detecting each individual, detection span, number of days an individual was detected, proportion of days detected, days detected/battery life, detection span/battery life, distance from tag location to 50% kernel centre, 50% kernel area (km^2^) and 95% kernel area (km^2^). These analyses allowed us to assess whether measurements of fish behaviour were biased or affected by non-biological technical characteristics of the tracking systems as well as their relationships to biological and ecological parameters.

### Long term residency

The proportion of animals remaining within the array after tagging was calculated using the detection span of each individual. When an animal was not detected within the array for more than one week, it was classified as having left the array. Animals that left the array and then returned were incorporated into the calculation at each time period. Two animals that were known to have been captured by recreational fishers and another two that were continuously recorded by one receiver were excluded from calculations at the date of capture or when the tag was only detected on one receiver. For comparisons of residency inside and outside the sanctuary zone, only data from animals tagged inside the lagoon were utilised as the Mangrove Bay Sanctuary does not extend past the reef crest.

## Results

### General trends in detection and kernel distribution

A total of 84 *L. nebulosus* ranging in size from 26–67 cm FL were tagged with acoustic tags within a large array of acoustic receivers (Appendix S1) and animals monitored for up to 864 days. Nine animals were not detected at all following release and the fate of these animals is uncertain. These nine animals were not included in subsequent analysis. The size range of animals tagged encompassed all sex and maturity stages, however the sex of tagged animals could not be determined. Animals were tagged at 34 locations on 17 occasions between 30/11/2007–6/11/2009 either in the lagoon or along the reef slope. This period spanned different intra-annual seasons and spawning periods. A range of habitats including, reef flats, lagoon coral outcrops and mangroves are present in the lagoon where fish were captured and tagged.

The results of our tagging program have revealed significant information relating to site fidelity and residency of individuals and also the variability in movement within the tagged population. Tagged *L. nebulosus* that were recorded at least once following release were detected up to 73,980 times on as many as 27 receivers over a maximum period of 891 days (Appendix S1). Throughout the detection period, over 70% of tagged fish were detected on less than 10 receivers (median = 5.0). Thirty nine of the 84 L. *nebulosus* that were tagged were detected over a period of 30 days, allowing a kernel distribution to be calculated (Appendix S1). Of these, two were thought to have died or been eaten close to a receiver resulting in the tag falling out of the animal and being continuously detected by one receiver only. Eight of the *L. nebulosus* for which kernels were calculated remained undetected for periods of several months following tagging. An additional 36 (48%) were detected following release but were detected for too short a time period and/or on too few receivers to warrant calculating kernel distributions. Although the kernel area for these 36 animals was not calculated, we have assumed that the distance from tag location to their centre of distribution was greater than 10 km. This assumption was based on the maximum distance between receivers on the edge of the array. Therefore animals that have moved greater than 10 km from any position within the array cannot be detected by receivers within the array.

Regression analyses demonstrated that the total number of detections (R-square = 0.044) and the span of the detection period (R-square = 0.124) were both significantly related to battery life which was expected. There was no significant relationship between the total number of days on which detections occurred and the range of parameters tested, however the proportion of days detected was significantly related to fish size and the habitat in which the fish was tagged. Although overall R-square was low (R-square = 0.14), larger fish tended to be detected on fewer days than smaller fish. Fish tagged in the lagoon were detected for longer periods than fish tagged on the reef slope (Table 1).

The number of receivers on which a fish was detected was significantly related to fish size (R-square = 0.14), with the largest fish tending to be detected on more receivers. The 50% and 95% kernel area were significantly related to habitat in which fish were tagged and explained far higher proportions of the total variability in the data (R-square of 0.42 and 0.67 respectively). Overall, fish tagged in the lagoon had smaller kernel area than fish tagged on the reef slope. Only a small amount of the variability (14%) in the 50% kernel area was explained by fish size (FL cm). Tag technical characteristics such as battery life, transmission interval and average detection range were not significant in regressions with kernel area.

### Kernel area and residence

**Figure 3:**
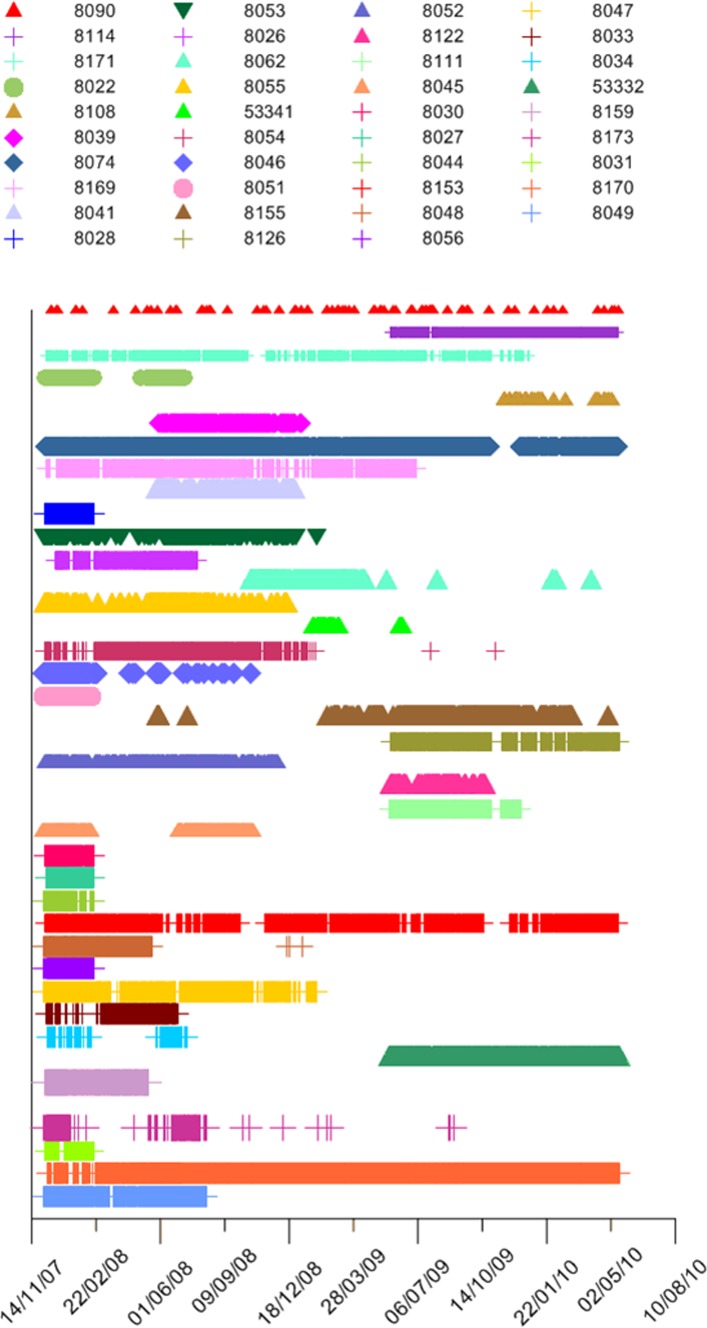
Detection plot of *Lethrinus nebulosus*. Detection plot of 39 *L. nebulosus* from date of tagging to last detection. Legend order from top left down equates to order of plot from top to bottom and matches order of Table 1.

**Figure 4:**
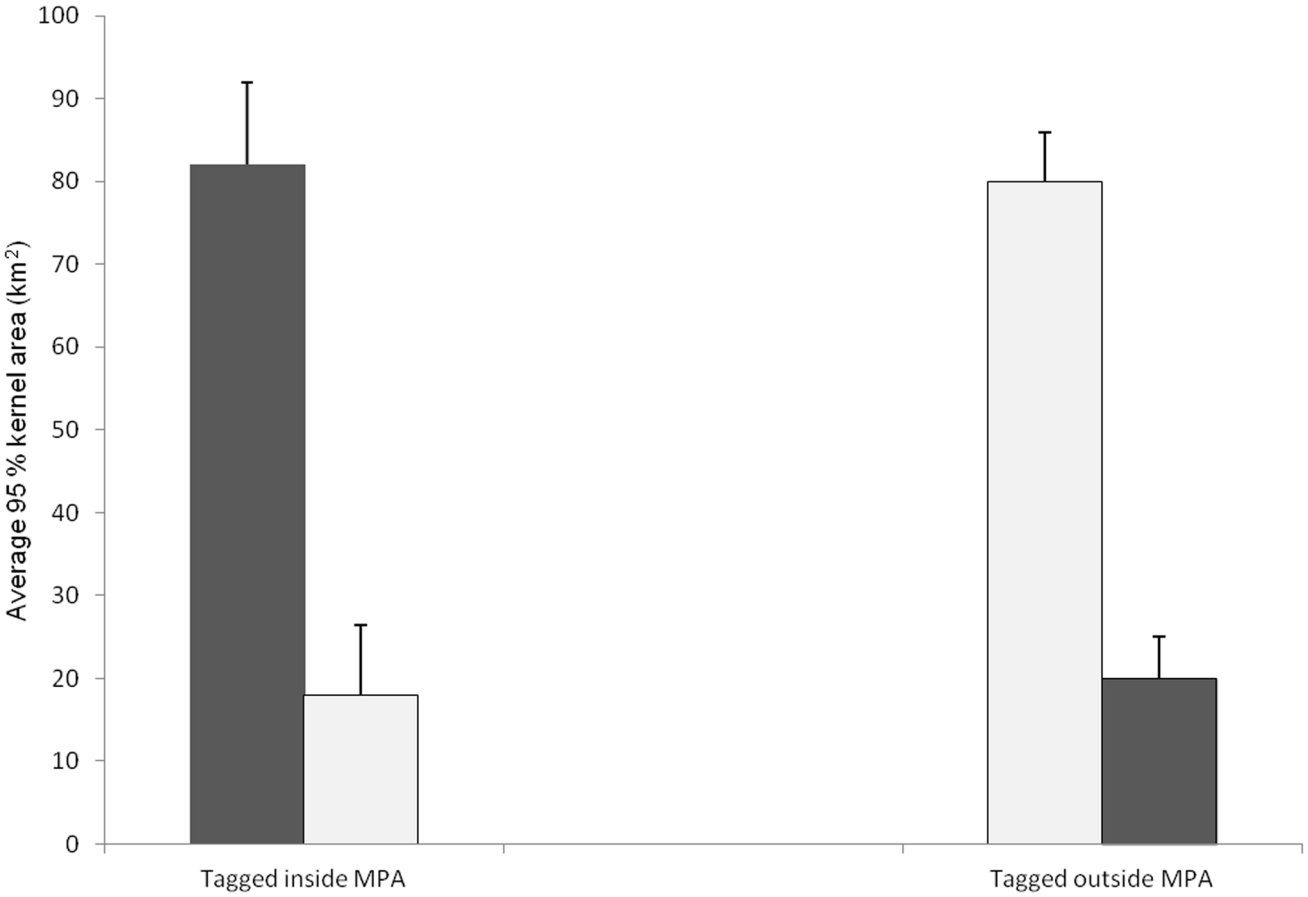
Kernel overlap with sanctuary zone. The average (+ SE) area of the 95% kernel distribution that occurred inside and outside the Mangrove Bay Sanctuary Zone for fish tagged within (n = 16) and outside (n = 23) the Sanctuary Zone boundary. Open bars: average 95% Kernel area inside MPA; dark bars: average 95% kernel outside MPA.

A detection history sufficient to allow calculation of kernel density distributions was obtained for 39 individuals (Figure 3) whose kernel distributions were calculated (Appendix S1). These 39 fish were detected regularly within the array with the median percentage of days detected being 78 (Appendix S1). The calculated 50 and 95% kernel areas ranged from 1.4–5.8 km^2^ (average 2.3±0.1 SE) and 4.9–21.1 km^2^ (average 8.6±0.6 SE) respectively. Given the large smoothing factor (1000) used to calculate KUD, these values are likely to be an overestimation of home range, but provide greater confidence in estimates of MPA effectiveness. Had we been attempting to estimate fine scale habitat use, these values would not be appropriate. There was no difference in the size of kernel area for fish tagged inside or outside the sanctuary zone boundary (Figure 4). For fish tagged inside the Mangrove Bay sanctuary zone (n = 16), the average proportion of the 95% kernel distribution that occurred within the sanctuary zone boundary was 81% (± 8 SE) with only a small degree of overlap (19%) with fished areas. Of those fish tagged outside the sanctuary (n = 23), the average proportion of the 95% kernel distribution that occurred inside the sanctuary zone boundary was 17% (± 6 SE) with the majority of kernel area within the fished area. Roughly half of all resident fish were detected on five receivers or less (Appendix S1). The remainder were detected on between 6–15 receivers with only a few individuals (10%) ranging more widely. This suggests that given the maximum of approximately 800 m separation between receivers, the vast majority of these fish use less than 5.8 km^2^.

Indeed, the majority of animals remained within the array during their detection period; however seven animals were detected frequently after tagging and were then not detected for periods varying from 47 to 209 days before again being detected. After the period of not being detected, all of these animals were detected on receivers where they had previously been detected, indicating a return to near the original location. Although fish 8046 was detected on the same receivers, it was also detected on a different suite of receivers approximately 3.0 km north of its previous activity centre.

Five of the fish that returned to the array were part of a group of 12 fish that ceased being detected between 5–22 February 2008. This period coincided with a Category three tropical cyclone “Nicholas” which crossed the Ningaloo coast on 19 February 2008. These five fish returned to the same area between the 13 April–27 June 2008. Seven fish were not detected again and presumably moved outside the array. The 12 fish that moved outside the array in February ranged in size from 26.5–49.5 cm FL with the returning fish 32.5–49.5 cm FL.

Seventeen fish were captured and tagged during spawning aggregations on the reef slope in December 2007, in the centre of the array adjacent to the reef pass. Following the end of the spawning season, the majority of these fish disappeared from the array, except for 8171 which returned to the lagoon where it remained for the next nine months before again moving offshore to the reef slope between October–December in 2008 and 2009. Of the fish that were not detected after 10 December 2007, several fish were detected by receivers on the reef slope in October, November and December in 2008 but not in 2009 whereas others were detected in 2009 but not 2008 suggesting that these fish returned to the same spawning site in some but not all years. Four fish tagged during spawning aggregations were detected on the line of cross shelf receivers off Tantabiddi (8074, 8111, 8165, 8171) with one fish (8139) also detected on receivers near Coral Bay. These individuals were recorded by the cross shelf lines immediately prior to and after the spawning season and were the only fish detected by receivers other than those within the Mangrove Bay array.

**Figure 5:**
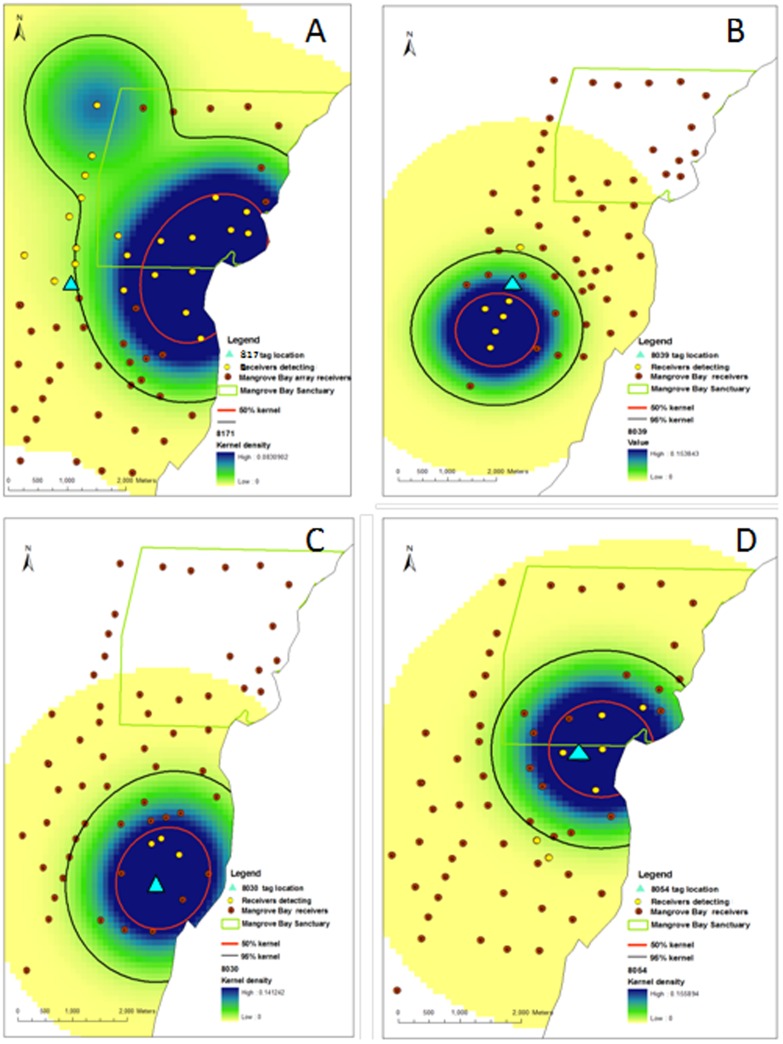
Kernel density of four individual *L. nebulosus*. Fixed kernel density of two *L. nebulosus* tagged on the reef slope (tag number 8171 (A), 8039 (B) and two tagged near coral outcrops in the lagoon 8030(C) and 8054 (D). The tagging location, receivers detecting the fish and all receivers within the array as well as the 50 and 95% kernel densities and fixed kernel density are shown. The boundary of the Mangrove Bay sanctuary is also shown.

A range of behaviours and patterns of habitat use were displayed by fish resident in the array. A 56 cm FL adult fish tagged during a November spawning aggregation returned to the lagoon in December 2007 and spent the rest of 2008 inside the lagoon until the following spawning season when it returned to the reef slope between October and December (Figure 5A). In contrast a 38.5 cm FL fish also tagged on the reef slope in May 2008 remained in the vicinity of its release over a period of 222 days (Figure 5B). This fish was one of a few fish on the reef slope that remained in the area where it was tagged for a long period of time. A 27 cm FL tagged in the lagoon in December 2007 was detected every day for 77 days before disappearing (Figure 5C)). This fish was one of a number of fish that departed the array in February 2008 (Figure 3) around the time of tropical cyclone “Nicholas”. During the same time period, a 34 cm FL fish tagged in December 2007 was detected over a period of 420 days (Figure 5D) until the tag battery presumably ran out as prior to 2010, VEMCO coded acoustic tags did not have a defined life span.

### Variability in behaviour and long term residency

**Figure 6:**
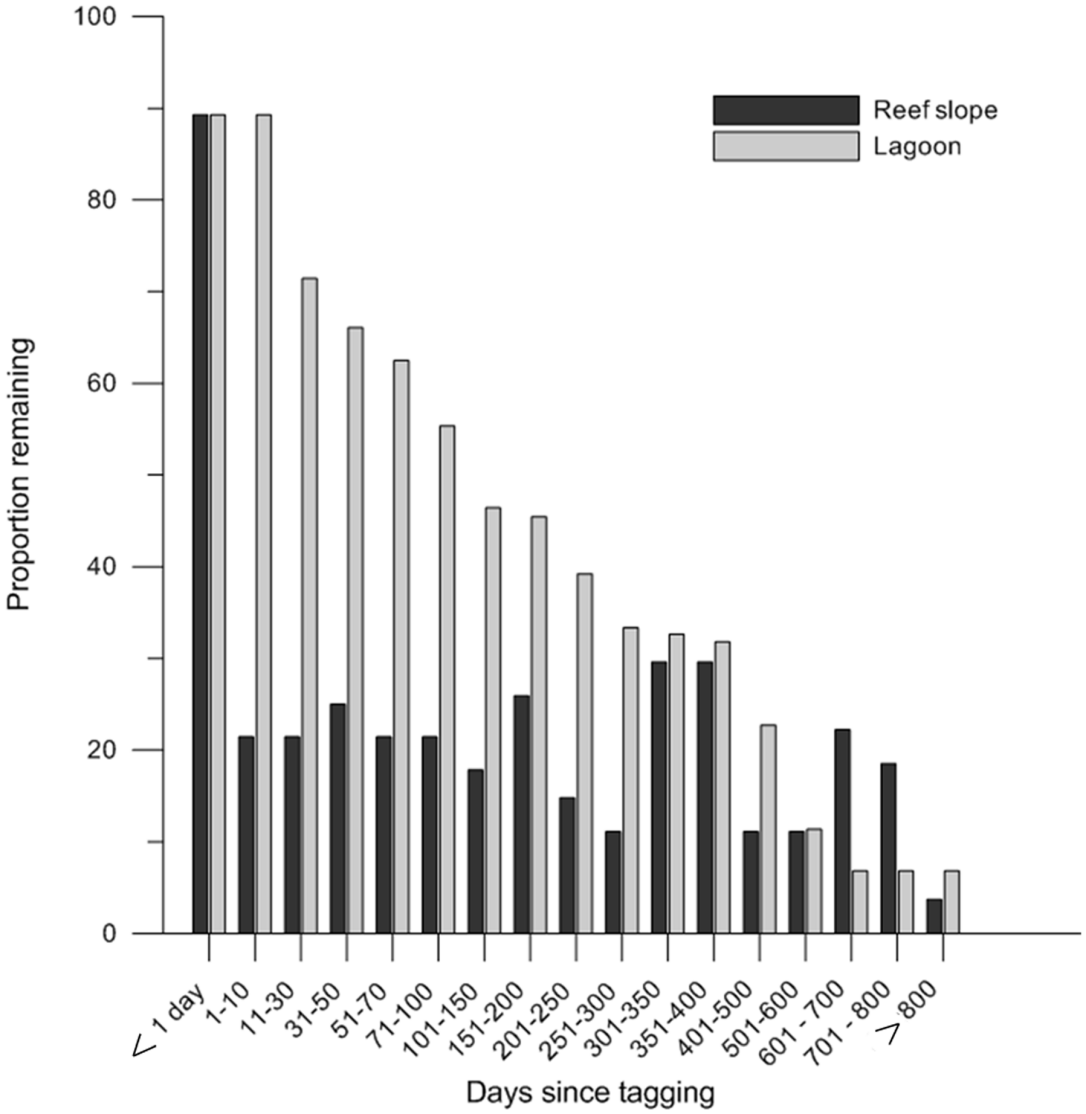
Long term decline in the number of individual fish remaining in the Mangrove Bay array. The percentage of *L. nebulosus* tagged in the lagoon and on the reef slope that were detected within the array at 1–800 days after tagging. The spawning period coincides with the time periods 301–350, 351–400 and 601–700 days since tagging.

The proportion of fish tagged in the lagoon and on the reef slope remaining within the array over time was determined from detection data (Figure 6). For all tagged fish (n = 84), there was a gradual decline in the number of animals that remained within the array. Fish tagged in the lagoon were more than twice as likely to remain within the array for long periods of time than fish tagged on the reef slope. For fish tagged in the lagoon, after 101–150 days and 301–350 days, 46% and 33%, respectively, were still being detected by the array. For fish tagged on the reef slope, 18% and 30% were still being detected after 101–150 and 301–350 days, respectively. The increase in the proportion of fish being detected on the reef slope after 301–400 days and again after 601–800 days was due to fish returning during the spawning season (October–December). When fish tagged on the reef slope were further divided into fish tagged during the spawning season and fish tagged outside the spawning season, fish tagged outside the spawning season were up to four times more likely to remain in the array for longer periods.

Long term residency was similar for fish tagged inside and outside the sanctuary zone. For fish tagged inside the sanctuary zone, after 101–150 days with 46% of those tagged inside the sanctuary and 36% of those tagged outside the sanctuary were still being detected. After 301–350 days, 27% of fish tagged inside and outside the sanctuary were still being detected.

**Figure 7:**
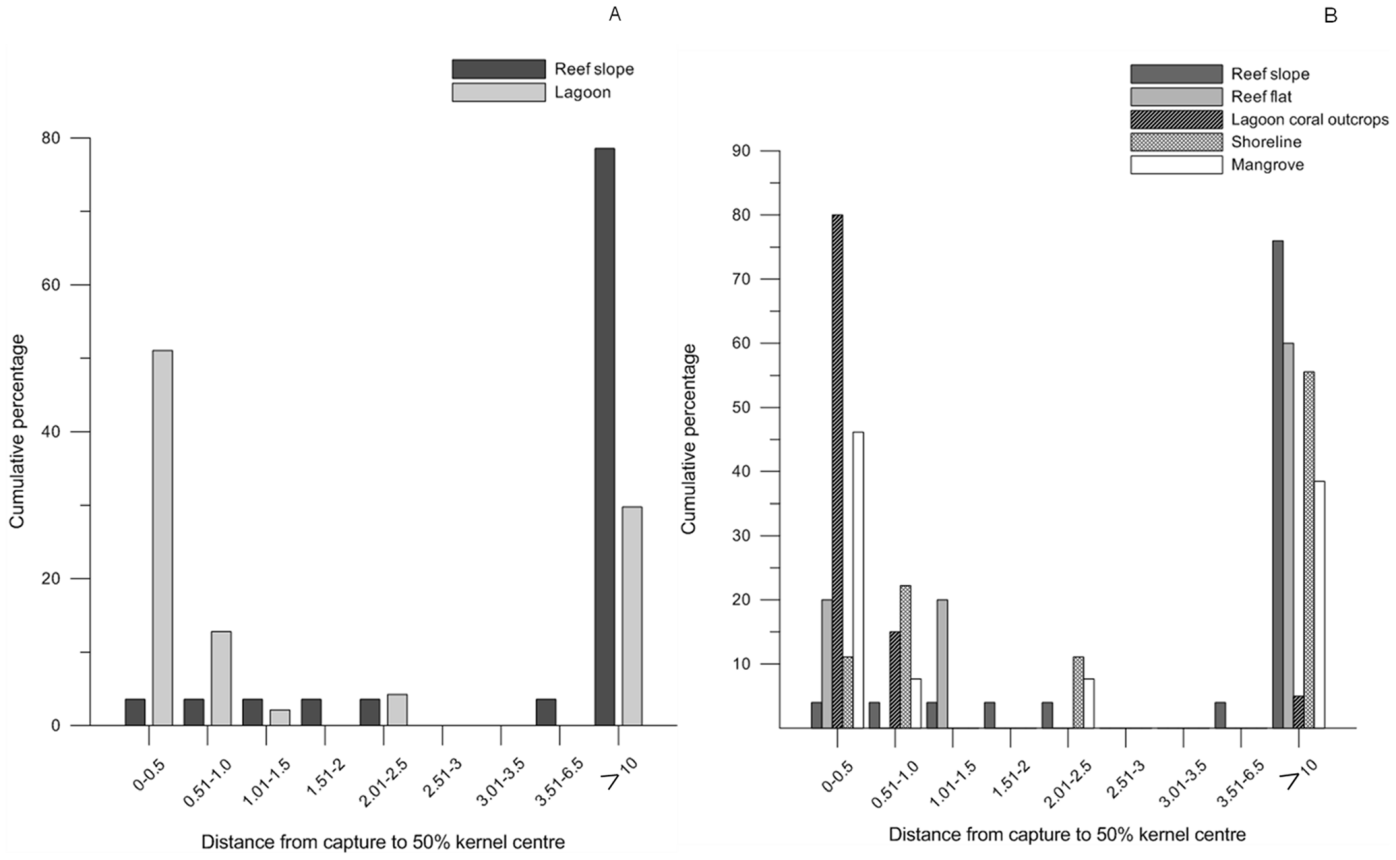
Distance from tag location to 50% kernel centre of 75 animals detected by the array of receivers. Cumulative percentage of distance from tag location to 50% kernel centre of 75 animals detected at some stage within the array. 7A) fish tagged on the reef slope and all lagoon habitats, 7B) reef slope and four different lagoon habitats. Sample size was 22, 7, 20, 9 and 16, respectively for reef slope, reef flat, lagoon coral outcrops, shoreline and mangrove.

Maximum distance moved between receivers was calculated for fish tagged inside the lagoon and on the reef slope. For animals tagged inside the lagoon, average (± SE) maximum distance moved was 2.92 (0.28) km which was significantly less (p = 0.009) than animals tagged on the reef slope 4.21 (0.46) suggesting that fish on the reef slope cover greater linear distances. For fish tagged inside the lagoon and on the reef slope, the average maximum distance moved did not differ significantly (p>0.6) between animals that were in the array for less than 30 days and animals that were detected for long periods suggesting that fish that moved out of the array were not pre-disposed to moving larger linear distances. Similarly, distance from position tagged to kernel centre was also smaller for animals tagged in the lagoon than those tagged on the reef slope (Figure 7A) as was average kernel area. When habitat-related trends of lagoon fish were examined in more detail it was clear that individuals associated with lagoon coral outcrops and mangroves had particularly high levels of site attachment, explaining much of the trend between lagoon and reef slope, since distances moved by reef flat and shoreline reef tagged individuals were more similar to those from fish tagged on the reef slope (Figure 7B).

## Discussion

Using acoustic telemetry we were able to examine the fine scale movements and residence behaviours of individual *L. nebulosus* within the NRMP over three years. Our results have implications both for the specifics of marine spatial management at the NRMP and also in how ecologists characterise home range, residency and spatial usage of reef dwelling marine fishes.

### Residency

The high resolution data gathered using acoustic telemetry revealed that *L. nebulosus* exhibit significant variability in their movement patterns and home range. Despite our methods resulting in home range being overestimated, a large proportion of the animals we tagged had a relatively small home range (average 95% kernel of 8.6±0.5 km^2^) and residency that could persist for more than 2.5 years. Long term stability in home range has been demonstrated in parrotfish *C. microrhinus* with home range persisting over 1.9 years [44] and *Scarus iserti* and *Sparismosa viride* with home range persisting for up to 3.5 years [45], [46]. However, despite the fact that some *L. nebulosus* remained in an area for several months to years, there is evidence of fish moving to areas outside the array with some returning to where they were tagged. However, several fish left the array entirely and did not return. These fish appear to be either nomadic without a defined home range or, more likely, have established home range/s outside the array. Chateau & Wantiez [47] demonstrated that *L. nebulosus* moved up to 1.7 km between reefs in New Caledonia and multiple activity centres have been described in *Pagrus auratus* [15].

To give these figures some context, our estimates of home range are 2–350 times greater than other coral reef species where data are available. Herbivorous unicorn fish (*Naso unicornis* and *N. litturatus*) have been shown to have a home range of 3.2–3.8 km^2^ in Guam [6] whereas steephead parrotfish (*C. microrhinus*) on the Great Barrier Reef had an average home range less than 1.0 km^2^ [27] which is up to 41 times greater than home range estimates of the surf parrotfish *Sparismosa viride* in the Caribbean [46]. Coral trout (*Plectropomus leopardus*) on the Great Barrier Reef had home ranges of 1.0–1.8 km^2^ [28] and a single Maori Wrasse had a home range of 5.0 km^2^ in New Caledonia [48]. In contrast more mobile green jobfish *Aprion viriscens* frequently moved more than 9.0 km between receivers [49] indicative of a home range several times larger than spangled emperor in the current study.

As noted in the methods, the characterisation of spatial utilisation from acoustic detection data is problematic. But despite the general shortcomings in the KUD method for characterising spatial distribution, our use of this approach should be robust to negatively biased estimates of home range size due to our choice of a large smoothing factor which should go some way to accounting for the detections arising from somewhere within a large radius around each receiver. Additionally our use of data only from individuals detected for more than 30 days means that the KUD estimates should be less prone to biases which might occur when individuals are observed for only very short or sporadic periods. The obvious downside of our method (and many others in current usage in similar studies) is that it does not permit formal statistical estimation of parameters describing the spatial distribution of individuals. Further investigation using recently developed state-space methods (e.g. [50]) may be useful in refining these estimates and will be investigated in future studies.

With respect to adequacy of the sanctuary zone size (areas where fishing is not permitted), the average size of sanctuary zones within the Ningaloo Marine Park is approximately 49 km^2^ however, over 70% are less than 30 km^2^ due to the large size of two of the sanctuary zones within the NRMP. The Mangrove Bay sanctuary is 11 km^2^ which is only slightly greater than the average 95% KUD for spangled emperor (8.5 km^2^). In the absence of long term monitoring and given that the dimensions of our array were 28 km^2^, this suggests that even some of the smallest sanctuary zones within the NRMP will provide some degree of protection for this species. However, after one year of monitoring, more than 60% of the fish tagged in the lagoon had moved beyond the detection range of the array of receivers. This indicates that sanctuary zones larger than 30 km^2^ are required to protect a significant proportion (>50%) of the population within the lagoon, as these fish tend to be less mobile than fish tagged on the reef slope.

### Variability in behaviour and long term residency

Our data indicate that while *L. nebulosus* populations include significant proportions of resident fish, making them a good candidate for MPA protection. The fact that many fish may be nomadic or utilise more than one core area suggests that networks of MPA's are likely to be necessary to protect a larger proportion of the population. Variability in movement behaviour within populations has also been described in other reef fish, such as *Pagrus auratus* and *Sebastes melanops* where between a third to more than half the population has been shown to exhibit high levels of movement [51], [52] or to have multiple activity centres [15]. Meyer et al. [5] demonstrated permanent emigration and excursions from Kealakekua Bay MPA by surgeonfish, parrotfish and goatfish providing evidence that relatively small reef fishes are less resident and more mobile than previously thought, although only 3% of all fish were detected moving two km or more from the point of capture. For *L. nebulosus*, it is not yet clear whether the contrast between residence at a single core area, as opposed to nomadism or occupation of multiple core areas represents two modes of behaviour, or a continuum of increasing home range size. A combination of additional telemetry data from an array of receivers significantly larger than that employed in this study combined with modelling approaches are required to better evaluate the network of MPA's within the NRMP.

Meyer et al. [5] showed that tagged reef fish relocated their home range within the array with individuals displaying shifts in space use and changes in the detection frequency at single receivers. However if multiple core areas exist for the *L. nebulosus* in our study they occur at scales either smaller than the range of a single receiver or larger than the Mangrove Bay array as there was no evidence of this in our study at the scales over which we were able to examine it. Nevertheless it is possible that individuals that left and then returned to the Mangrove Bay array had shifted their core areas displaying multiple activity centres. One returning fish (8046) appeared to have shifted core areas.

The departure of 12 fish in February 2008 coincided with the approach of a Category three tropical cylone “Nicholas”. It is possible that the weather conditions and falling barometric pressure associated with this cyclone caused some individuals to move to deeper waters. A similar response was demonstrated by young of the year Blacktip Sharks (*Carcharhinus limbatus*) in Florida where all 13 animals being monitored by an array of receivers departed the shallow waters of Terra Ceia Bay a few hours before the arrival of a tropical storm and then returned several hours after the storm had passed [53]. The departure and return of these Blacktip sharks was very closely aligned to the arrival and departure of the storm, in contrast to the behaviour of juvenile and adult *L. nebulosus* that departed over a longer time period. Similarly, fish that returned did so over a period of several weeks to four months.

Fisheries based estimates of mortality for *L. nebulosus* in the region range between Z = 0.285–0.384 [30] which would mean that between 57 and 45% of tagged individuals would be expected to survive for two years after tagging. While these are probably minimum estimates (being derived from populations outside protected areas), the number of tagged animals remaining in the array after two years was much lower than these values (Figure 6). Emigration from the study site is therefore considered likely to be the largest component of declining number of animals detected over time, with declining numbers being a net product of both departures and returns of tagged individuals.

Previous studies have demonstrated declining numbers of detections in tagged cohorts beginning shortly after tagging and continuing to decline throughout the monitoring period [5], [25], [54], [55], [56]. These trends have been evident within time spans significantly less than expected battery life. The interpretation of such declines is problematic and they have been attributed to a combination of the following: 1) premature transmitter failure, 2) tagging effects (elevated predation, increased mortality or transmitter expulsion, 3) mortality unrelated to tagging (natural of fishing mortality) and 4) emigration from the study site [5]. Viewed in aggregate the residency patterns of *L. nebulosus* at Mangrove Bay appear to produce a picture that resembles the result of dispersive processes (Figure 6) with the number of tagged individuals in the array gradually declining, and we consider this the likely explanation for the majority of the decline. There was some evidence of fishing mortality at Mangrove Bay with two animals recaptured by recreational fishers (as demonstrated also by [25], [54], [55], [56]). There was also evidence of natural mortality; characteristic patterns of tag detections are recorded where animals either died or were consumed close to a receiver resulting in the tag falling out and being continually detected by one receiver. There was also one instance where an animal displayed limited movement over a period of months, followed by weeks of large scale roaming within the array until the tag was detected continuously on one receiver. This was consistent with movement patters of black tip reef sharks (*Carcharhinus melanopterus*) tagged as part of this study and we suspect this individual was consumed by a black tip reef shark. Eventual eversion of the stomach is likely to have caused the tag to fall to the bottom near a receiver as it is common for carcharhinid sharks to periodically evert their stomachs completely into the oral cavity, possibly to expel undigested food or gastric parasites [57].

### Effects of zoning and implications for reserve design

It is clear that approximately half the *L. nebulosus* population in the Mangrove Bay sanctuary zone at any time receives any lasting protection from it. But despite the high residency shown by *L. nebulosus* in Mangrove Bay over short periods (weeks to months), our long-term movement data illustrate that even these resident fish will have a greater than 20% chance of crossing the reserve boundary (Figure 6). For non-resident fish the likelihood of spending time outside the sanctuary zones is of course even greater. While home range size of tracked fish is significantly smaller than the average MPA size within the Ningaloo Reef Marine Park, both maximum linear distance moved by fish within the array as well as evidence of a large proportion of fish moving beyond the limits of the acoustic array suggest large sanctuary zones will be needed to protect a larger proportion of the population within the NRMP. Surveys of the NRMP have shown higher biomass of *L. nebulosus* in no-take zones [31], [58] but it has not been possible to date to demonstrate a relationship between reserve size and *L. nebulosus* abundance. For sanctuary zones established in 1986 neither reserve size (area, perimeter, sea perimeter (perimeter excluding the terrestrial and shoreline boundary), area/perimeter and area/sea perimeter) were correlated with the relative abundance of *L. nebulosus* [31].

Results of the current study showed that fish tagged on the reef slope displayed greater levels of movement than fish tagged in the lagoon, with fish on the reef slope more likely to move outside the monitored area. These movement data are supported by surveys of *L. nebulosus* biomass within the NRMP. Biomass was higher inside the lagoon than on the reef slope with significantly higher biomass inside protected areas within the lagoon than adjacent fished areas [31]. In contrast, biomass of *L. nebulosus* on the reef slope was higher in fished areas than protected areas. Although the array could detect long-shore movement of fish on the reef slope, it was not primarily designed to detect offshore movements with the deepest receivers at approximately 50 m depth and two km from the reef crest. Movement of fish on the reef slope to deep water habitat more than 10 km offshore therefore requires additional research.

There are several lines of evidence consistent with the conclusion that the sizes of many sanctuary zones at Ningaloo are too small to achieve conservation outcomes (i.e. maintain or restore impacted populations). The response to no-take status in terms of effect size (no-take:fished biomass) is relatively small (1.5 times greater biomass), even for sanctuary zones established in 1986 [31] and at the one site where such comparisons are possible, the abundance of *L. nebulosus* has declined several fold both inside and outside the Osprey Sanctuary Zone [31]. Using an age-based demographic analysis of fish captured along the Ningaloo Reef in 1989–1991 and 2007–2008, Marriott et al. [33] showed that under 2007–2008 fishing levels, *L. nebulosus* were at risk of overfishing. Furthermore, levels of fishing pressure have had a dramatic effect on fish stocks in this region with the modal age of fish declining from six to five years and the percentage of fish older than six years declined from ∼50% in 1989–1991 to ∼28% in 2007–2008.

The Mangrove Bay Sanctuary zone is one of the smaller no-take zones in the Ningaloo Marine Park and, for resident *L. nebulosus*, our results show that such a no-take zone can offer reasonably high levels of protection. However, at least 48% of fish moved distances great enough to take them not only out of the reserve but out of the array altogether.

Differences in residence times between the lagoon and reef slope are likely to be due to the fact that animals tagged on the reef slope during spawning periods had moved to this area as part of a spawning migration. There was evidence of this with one fish returning to the lagoon after the spawning season and remaining within the lagoon for 10 months before moving offshore in October–December. Similarly, several adult fish tagged during the spawning season were only recorded by the array between October–December and then only on receivers on the reef slope. Five sexually mature fish tagged during spawning aggregations were detected on lines of cross shelf receivers during spawning periods (October–December). Four fish were detected off Tantabiddi (∼10 km north) and one off Coral Bay (∼80 km south). These detections suggest fish undergo long distance movements associated with spawning that are significantly greater than home range estimates. Long distance spawning movements are common even in highly site attached species such as coral groupers [59]. The scale and frequency of these movements requires additional research. Unfortunately these lines of cross-shelf receivers did not extend into the lagoon preventing us detecting large scale movements within the lagoon.

Fish tagged on the reef slope had a greater average kernel area, moved greater linear distances between receivers than fish tagged inside the lagoon. These differences are most likely due to the habitat on the reef slope being confined to a narrow band from the reef crest to ∼30 m. This may result in animals either having a longer home range or shifting home range more frequently. For coral trout in the GBR, reef width accounted for 65% of the variability in home range area for animals on fringing reefs, whereas individuals tracked around patch reefs had a larger home range due primarily to the width of estimated home range [24].

Higher rates of movement and larger activity centres of individual *L. nebulosus* in reef slope habitats, and the apparent targeted use of this habitat for spawning by at least some of the population, indicates the need for increased size of no-take zones in these habitats is even greater. But the reef slope at Mangrove Bay is not protected by a no-take zone, a situation that is typical of most sanctuary zones in the northern section of the NMP where deeper reef habitats are under-represented.

### Influence of tag parameters

The battery life, transmission interval and measured range of tags in this study varied from 185–2020 days, 40–320 s and 50->500 m, respectively. That there was no relationship between battery life, tag transmission interval and average tag range and ecological measurements such as kernel size suggests that biological and ecological factors are by far the most important factors in determining the dimensions of fish activity centres in our tracking data. Furthermore, the configuration of the array (as indexed by distance to array edge) was not a significant parameter in any of the regressions, suggesting that our conclusions are not biased by the size of the array or the location of tagging with respect to the array location. These findings have important implications for the design of acoustic arrays and suggest that the results of tagging studies are extremely robust and that the behaviour of individual fish is far more important than the behaviour or characteristics of tags used to monitor individuals. We did not find any indication that the non-overlapping nature of the receivers in the array detracted from its effectiveness in informing our understanding of the movements of *L. nebulosus*.

There is an increasing body of literature on the movement and home range of reef fish that attempts to relate tracking data to MPA effectiveness; this has resulted in considerable variation in the species studied, spatial and temporal scale, study duration and sampling methods used to generate movement data. While there is mounting evidence that the majority of coral reef fish remain within a small core area, studies are commonly limited to few individual animals of the same size and/or, that are monitored by few receivers (normally fewer than 10) that have limited coverage of available habitat (usually less than 5 km^2^) and have been conducted for a short time period (3–6 months) [5], [25], [27], [60], [61], [62]. For example, a study of *C. microrhinus* tracked animals for less than five days and described home ranges as having stabilised after 3–4 days with no evidence of home range increasing over time [27]. If such short time frames had been applied to our studies of *L. nebulosus* we would have come to quite different conclusions to those we have drawn over a period of years.

While we acknowledge the usefulness of these studies and the fact that battery life, tag size and cost have increased the ability to conduct long term studies on larger numbers of fish, it is important to be aware that important variability exists within populations at a range of spatial, temporal and ontogenetic levels. Management decisions made on the basis of relatively limited and short term observations may be compromised if they do not include the full range of information required for application at a population level. This is particularly true in the case of designing reserves for properties such as “spillover” which is by definition a complex product of movements into and out of no-take areas, growth, recruitment, reproduction and fishing behaviours which needs to be modelled [16] to assess the likelihood of effective net results on either side of the conservation/fisheries ledger.

### Conclusions

The behaviour of *L. nebulosus* within the Ningaloo Reef Marine Park was variable and complex. The majority of individuals had home ranges that were smaller than current MPA's, however after one year of monitoring more than half the fish had moved beyond the monitored area and outside the MPA boundary. Within NRMP, MPA's may need to be expanded to offer greater protection to the species, particularly with respect to fish on the reef slope that were more mobile. Furthermore, annual spawning aggregations adjacent to reef passes have important implications for MPA design and planning.

## Supporting Information

Appendix S1 Tagging and detection details for all fish.
(DOCX)
