# Correction: Multi Year Observations Reveal Variability in Residence of a Tropical Demersal Fish, *Lethrinus nebulosus*: Implications for Spatial Management

**DOI:** 10.1371/journal.pone.0118869

**Published:** 2015-03-05

**Authors:** The PLOS ONE Staff

There is an error in the fourth author’s name. The correct name is Ryan A. Downie. The correct citation is: Pillans RD, Bearham D, Boomer A, Downie RA, Patterson TA, Thomson DP, et al. (2014) Multi Year Observations Reveal Variability in Residence of a Tropical Demersal Fish, *Lethrinus nebulosus*: Implications for Spatial Management. PLoS ONE 9(9): e105507. doi:10.1371/journal.pone.0105507
