# Supplementary material for: Multi Year Observations Reveal Variability in Residence of a Tropical Demersal Fish, Lethrinus nebulosus: Implications for Spatial Management
Source: PLoS One. 2014 Sep 2;9(9):e105507. doi: 10.1371/journal.pone.0105507 (PMC4152176; doi:10.1371/journal.pone.0105507)
Supplement: Appendix S1 — Tagging and detection details for all fish. (DOCX) [file pone.0105507.s001.docx]

**Appendix S1. Tagging and detection details for all fish.**

| Tag ID | FL cm | Date | Battery life | Total detections | Receivers detected on | Detection span | Days detected | % days detected | Distance to kernel centre | 50% kernel area | 95% kernel area |
| --- | --- | --- | --- | --- | --- | --- | --- | --- | --- | --- | --- |
| 8090 ^b, f^ | 51 | 28-May-08 | 1470 | 81 | 3 | 882 | 75 | 9 | 6.07 | 2.4 | 14.78 |
| 8114** ^a^ | 56 | 23-May-09 | 1470 | 132691 | 24 | 354 | 351 | 99 | 2.5 | 1.5 | 6.64 |
| 8171 ^f^ | 56 | 6-Dec-07 | 2020 | 11370 | 23 | 751 | 565 | 75 | 2.5 | 3.43 | 16.6 |
| 8022 ^c^ | 37 | 4-Dec-07 | 185 | 5095 | 10 | 217 | 123 | 57 | 2.14 | 1.79 | 7.67 |
| 8108 ^e^ | 54.5 | 4-Nov-09 | 1470 | 235 | 13 | 168 | 31 | 18 | 1.52 | 5.84 | 21.09 |
| 8039 ^a^ | 38.5 | 29-May-08 | 185 | 1630 | 7 | 222 | 180 | 81 | 1.06 | 2.3 | 9.93 |
| 8074 ^e^ | 48 | 4-Dec-07 | 820 | 48052 | 27 | 890 | 803 | 90 | 0.74 | 2.7 | 9.07 |
| 8169 ^a^ | 46 | 5-Dec-07 | 2020 | 10256 | 13 | 576 | 490 | 85 | 0.7 | 1.96 | 6.94 |
| 8041 ^a^ | 34 | 25-May-08 | 185 | 716 | 7 | 214 | 135 | 63 | 0.64 | 2.36 | 10.54 |
| 8028 ^d^ | 28 | 4-Dec-07 | 185 | 9666 | 4 | 77 | 77 | 100 | 0.62 | 2.33 | 7.85 |
| 8053 ^b^ | 35.5 | 3-Dec-07 | 353 | 920 | 4 | 424 | 200 | 47 | 0.57 | 2.43 | 9.71 |
| 8026 ^b^ | 39.5 | 3-Dec-07 | 185 | 7148 | 9 | 220 | 201 | 91 | 0.53 | 1.84 | 6.8 |
| 8062 ^a^ | 51.5 | 20-Oct-08 | 820 | 36731 | 6 | 529 | 177 | 33 | 0.5 | 1.42 | 5 |
| 8055 ^a^ | 38 | 3-Dec-07 | 353 | 572 | 5 | 376 | 145 | 39 | 0.5 | 2.45 | 9.6 |
| 53341 ^e^ | 42.5 | 23-Jan-09 | 520 | 2842 | 7 | 140 | 37 | 26 | 0.44 | 2.38 | 9.42 |
| 8054 ^a^ | 34 | 3-Dec-07 | 353 | 3427 | 6 | 420 | 359 | 85 | 0.42 | 1.88 | 7.42 |
| 8046 ^e,b^ | 40 | 2-Dec-07 | 353 | 2541 | 10 | 322 | 96 | 30 | 0.4 | 2.27 | 8.59 |
| 8051 ^d^ | 36 | 2-Dec-07 | 353 | 1045 | 2 | 78 | 71 | 91 | 0.35 | 2.49 | 9.49 |
| 8155 ^a^ | 43 | 27-May-08 | 2020 | 46015 | 4 | 700 | 291 | 42 | 0.3 | 2.13 | 9.15 |
| 8126 ^f^ | 53 | 23-May-09 | 1470 | 21151 | 11 | 357 | 312 | 87 | 0.3 | 1.66 | 6.42 |
| 8052 ^a^ | 37 | 3-Dec-07 | 353 | 1912 | 6 | 602 | 294 | 49 | 0.3 | 2.35 | 9.36 |
| 8122 ^a^ | 50 | 23-Jan-09 | 1470 | 1028 | 1 | 146 | 84 | 58 | 0.3 | 1.42 | 4.95 |
| 8111 ^f^ | 55 | 23-May-09 | 1470 | 15697 | 11 | 205 | 190 | 93 | 0.28 | 1.72 | 6.43 |
| 8045 ^c^ | 39 | 1-Dec-07 | 353 | 2647 | 4 | 327 | 181 | 55 | 0.25 | 2.2 | 8.12 |
| 8030 ^d^ | 27 | 4-Dec-07 | 185 | 4342 | 3 | 77 | 77 | 100 | 0.23 | 2.43 | 8.43 |
| 8027 ^d^ | 26.5 | 5-Dec-07 | 185 | 6986 | 4 | 74 | 74 | 100 | 0.22 | 1.8 | 7.28 |
| 8044 ^d^ | 32 | 1-Dec-07 | 353 | 1360 | 2 | 78 | 64 | 82 | 0.21 | 2.18 | 7.96 |
| 8153 ^f^ | 53 | 30-Nov-07 | 1470 | 73980 | 18 | 891 | 731 | 82 | 0.2 | 2.42 | 10.45 |
| 8048 ^e^ | 41 | 3-Dec-07 | 353 | 13978 | 4 | 402 | 173 | 43 | 0.2 | 2.21 | 8.78 |
| 8056 ^d^ | 33 | 3-Dec-07 | 353 | 2873 | 5 | 78 | 77 | 99 | 0.17 | 2.21 | 7.93 |
| 8047* ^a^ | 41 | 3-Dec-07 | 353 | 5940 | 4 | 424 | 353 | 83 | 0.16 | 2.17 | 7.88 |
| 8033 ^a^ | 37 | 6-Dec-07 | 185 | 2751 | 3 | 204 | 146 | 72 | 0.16 | 2.22 | 8.08 |
| 8034 ^c^ | 32.5 | 6-Dec-07 | 185 | 2381 | 4 | 217 | 79 | 36 | 0.16 | 2.15 | 8.09 |
| 53332 ^a^ | 45 | 23-May-09 | 520 | 24111 | 11 | 357 | 349 | 98 | 0.15 | 1.55 | 5.74 |
| 8159* ^a^ | 47 | 4-Dec-07 | 2020 | 7954 | 10 | 411 | 162 | 39 | 0.15 | 1.6 | 7.16 |
| 8173 ^c, b^ | 49.5 | 3-Dec-07 | 2020 | 6580 | 7 | 636 | 110 | 17 | 0.14 | 1.82 | 5.86 |
| 8031 ^d^ | 26.5 | 4-Dec-07 | 185 | 4384 | 4 | 76 | 71 | 93 | 0.14 | 2.18 | 7.95 |
| 8049 ^a^ | 38 | 3-Dec-07 | 353 | 9152 | 1 | 253 | 244 | 96 | 0.15 | 2.51 | 7.78 |
| 53270 | 26 | 23-Jan-09 | 773 | 627 | 3 | 15 | 15 | 100 | NA | NA | NA |
| 8170** | 45 | 5-Dec-07 | 2020 | 284316 | 12 | 889 | 864 | 97 | NA | NA | NA |
| 53238 | 27 | 23-Jan-09 | 425 | 1464 | 1 | 436 | 168 | 39 | NA | NA | NA |
| 8078 | 56 | 1-Dec-07 | 820 | 966 | 5 | 347 | 8 | 2 | NA | NA | NA |
| 8197 | 61 | 6-Nov-09 | 480 | 741 | 22 | 216 | 6 | 3 | NA | NA | NA |
| 8140 | 60 | 6-Nov-09 | 1470 | 368 | 11 | 161 | 15 | 9 | NA | NA | NA |
| 8036 | 34 | 5-Dec-07 | 185 | 344 | 24 | 11 | 11 | 100 | NA | NA | NA |
| 8198 | 58.5 | 30-May-08 | 480 | 319 | 4 | 8 | 8 | 100 | NA | NA | NA |
| 8032 | 37 | 27-May-08 | 185 | 298 | 5 | 20 | 15 | 75 | NA | NA | NA |
| 53280 | 26 | 23-May-09 | 773 | 229 | 5 | 15 | 15 | 100 | NA | NA | NA |
| 8025 | 41 | 4-Dec-07 | 185 | 223 | 2 | 107 | 9 | 8 | NA | NA | NA |
| 8199 | 67 | 30-May-08 | 480 | 171 | 11 | 30 | 5 | 17 | NA | NA | NA |
| 8168 | 57 | 6-Dec-07 | 2020 | 158 | 23 | 754 | 12 | 2 | NA | NA | NA |
| 8094 | 56.5 | 25-May-08 | 1470 | 134 | 4 | 60 | 10 | 17 | NA | NA | NA |
| 8120 | 47.5 | 23-Jan-09 | 1470 | 117 | 6 | 3 | 3 | 100 | NA | NA | NA |
| 8167 | 56 | 6-Dec-07 | 2020 | 90 | 3 | 1 | 1 | 100 | NA | NA | NA |
| 8164 | 51 | 6-Dec-07 | 2020 | 87 | 11 | 342 | 4 | 1 | NA | NA | NA |
| 8165 | 54 | 6-Dec-07 | 2020 | 81 | 19 | 737 | 7 | 1 | NA | NA | NA |
| 8100 | 58 | 5-Nov-09 | 1470 | 60 | 14 | 55 | 5 | 9 | NA | NA | NA |
| 8163 | 57 | 6-Dec-07 | 2020 | 56 | 13 | 737 | 6 | 1 | NA | NA | NA |
| 8154 | 44 | 3-Dec-07 | 2020 | 52 | 5 | 4 | 4 | 100 | NA | NA | NA |
| 8095 | 59 | 6-Dec-07 | 1470 | 48 | 15 | 361 | 6 | 2 | NA | NA | NA |
| 8024 | 34 | 4-Dec-07 | 185 | 42 | 7 | 6 | 6 | 100 | NA | NA | NA |
| 8103 | 53 | 3-Dec-07 | 1470 | 41 | 1 | 57 | 10 | 18 | NA | NA | NA |
| 8166 | 56 | 6-Dec-07 | 2020 | 21 | 11 | 362 | 5 | 1 | NA | NA | NA |
| 8162 | 67 | 6-Dec-07 | 2020 | 17 | 12 | 739 | 4 | 1 | NA | NA | NA |
| 8105 | 61 | 23-May-09 | 1470 | 17 | 1 | 1 | 1 | 100 | NA | NA | NA |
| 8050 | 34 | 3-Dec-07 | 353 | 15 | 6 | 248 | 11 | 4 | NA | NA | NA |
| 8188 | 56.5 | 5-Nov-09 | 480 | 10 | 3 | 89 | 2 | 2 | NA | NA | NA |
| 8023 | 28.5 | 4-Dec-07 | 185 | 10 | 2 | 9 | 4 | 44 | NA | NA | NA |
| 8158 | 49 | 3-Dec-07 | 2020 | 10 | 1 | 5 | 3 | 60 | NA | NA | NA |
| 8160 | 56 | 6-Dec-07 | 2020 | 8 | 5 | 1 | 1 | 100 | NA | NA | NA |
| 8043 | 31.5 | 1-Dec-07 | 353 | 6 | 2 | 250 | 2 | 1 | NA | NA | NA |
| 8038 | 37 | 26-May-08 | 185 | 5 | 2 | 364 | 5 | 1 | NA | NA | NA |
| 53340 | 46 | 23-Jan-09 | 520 | 4 | 1 | 1 | 1 | 100 | NA | NA | NA |
| 8072 | 38.5 | 27-May-08 | 820 | 2 | 1 | 1 | 1 | 100 | NA | NA | NA |
| 8157 | 43 | 5-Dec-07 | 2020 | 1 | 1 | 1 | 1 | 100 | NA | NA | NA |

**Legend:** Tag number, size, date tagged and battery life of 84 *L. nebulosus* tagged between November 2007 – November 2009. Total number of detections, number of receivers each individual was detected on, detection span (date from first detection to last detection), days detected (total number of days each individual was detected on) and percentage of days recorded (days detected/detection span * 100) are shown. The distance from tag location to the 50% kernel centre, 50 % and 95% kernel area are also shown.

* Fish 8047 and 8159 were recaptured by recreational fishers very close to the array on the 1/7/2009 and 15/5/2008, respectively. **Fish 8114 and 8170 were moving around within the array until the 5/8/09 and 4/7/08, respectively. After these dates, the tag was stationary near one receiver. Kernel data for these two fish was only calculated from data prior to the tag becoming stationary.

^a^ long term residents detected frequently throughout their residency

^b^ long term residents detected infrequently at some period during their residency

^c^ resident fish that departed in February 2008 and returned during 2008

^d^ resident fish that departed outside of February 2008 and returned

^e^ resident fish that departed in February 2008 and didn’t return

^f^ long term residents that were not detected during the spawning season (October – December) in one or more years
